# The Effects of Phytosterols Extracted from *Diascorea* *alata* on the Antioxidant Activity, Plasma Lipids, and Hematological Profiles in Taiwanese Menopausal Women

**DOI:** 10.3390/nu9121320

**Published:** 2017-12-05

**Authors:** Chao-Chin Hsu, Hsin-Chih Kuo, Ko-En Huang

**Affiliations:** 1 Graduate Institute of Medical Science, Chang Jung Christian University, Tainan 71101, Taiwan; 2 Department of Obstetrics and Gynecology, Taipei Medical University Hospital, Taipei 110, Taiwan; 3 Department of Health Management, I-Shou University, Kaohsiung 84001, Taiwan; simon9704@gmail.com; 4 Department of Obstetrics and Gynecology, Chang Gung University School of Medicine and Kaohsiung Chang Gung Memorial Hospital, Kaohsiung 83301, Taiwan; 5 San an Obstetrics and Gynecology Hospital, 177 Meisu East 2nd Road, Kaohsiung 804, Taiwan

**Keywords:** *Diascorea*, phytosterol, menopause, hematological profiles, lipid profile, antioxidant activity, breast cyst

## Abstract

The efficacy of phytosterols extracted from *Diascorea alata* on antioxidant activities, plasma lipids and hematological profiles was assessed in postmenopausal women. Gas chromatography and mass spectrophotometry was employed to determine the steroid content of Taiwanese yam (*Diascorea alata* cv. Tainung No. 2). A two-center, randomized, double-blind, placebo-controlled clinical investigation on 50 postmenopausal women randomly assigned to two groups treated for 12 months with placebo or two sachets daily of *Diascorea* extracts containing 12 mg/dose was carried out. The main outcome measures were the plasma antioxidant activities, hematological profiles, and the concentrations of plasma lipids, including cholesterol, triglyceride, low density lipoprotein, high density lipoprotein, very low density lipoprotein,, and apolipoprotein A1 and B. A one-way analysis of covariance (ANCOVA) test was performed to investigate the significance. Beta-sitosterol, stigmasterol, 22-23-dihydro-, and γ-sitosterol were major phytosterols determined from *Diascorea* extracts. At six months in those receiving *Diascorea*, there were significantly decreased leukocyte counts (*p* < 0.01) and improvement on antioxidant activity of malondialdehyde (*p* < 0.001). After 12 months’ treatment, elevations of hematocrit and mean corpuscular volume (*p* < 0.01) were noted in those receiving *Diascorea*. Moreover, the low dose *Diascorea* consumption in menopausal women for one year generally did not present positive effects on lipid profiles.

## 1. Introduction

Mounting evidence indicates that, as women age and enter menopause, they undergo significant changes in cardiovascular risk factors, including adverse alterations in lipids, blood pressure, body fat distribution, oxidative stress, and cardiometabolic parameters [1,2]. Free radicals, reactive oxygen species (ROS), are produced either from the aging processes or from external sources, such as exposure to radiation, cigarette smoking, air pollutants, and industrial chemicals. The oxidation induced by ROS may result in cell membrane disintegration, membrane protein damage and DNA mutations, which play an important role in aging and the development of many diseases, such as arteriosclerosis, cancer, diabetes mellitus, liver injury, inflammation, coronary heart diseases, and arthritis [3,4,5].

Aging is associated with a number of qualitative changes in hematopoiesis, including decreased self-replicating ability of hematopoietic stem cells (HSCs), reduced responsiveness of HSCs to growth factor, reduced activity of neutrophils, monocytes, dendritic cells, and some lymphocyte subsets [6,7]. Estrogenic hormone has been implicated as an inhibitor of erythropoiesis and erythropoietin induction [8,9]. A diminished level of estrogenic hormone in menopause has been associated with increased hemoglobin levels [10]. 

*Diascorea* yam (*Diascorea* spp.) is an important tuber for the promotion of health and longevity in Chinese tradition [11]. *Diascorea alata* as a staple food for 30 days has been shown to decrease plasma cholesterol levels, and the prolonged lag time of low density lipoprotein oxidation. These effects might potentially protect postmenopausal women against the risk of breast cancer and cardiovascular diseases [12]. Our previous study indicated that *Diascorea* treatment can effectively improve the psychological parameters in healthy Taiwanese postmenopausal women [13]. There was dramatic increased usage of alternative therapy, including herbs and botanical ingredients, in menopausal women after a World Health Organization report in 2002 [14]. This raised great concern to the safety of herbs and botanical preparations, as little is known about their active ingredients [15]. In the present study, the phytosteroids contents of *Diascorea alata* was determined using gas chromatography and mass spectrophotometry (GC-MS). We examined, for the first time, the effects of plant sterols through *Diascorea* consumption on antioxidant activity, lipid, and hematological profiles in postmenopausal women.

## 2. Materials and Methods

### 2.1. Reagents

All reagents were analytical grade. Cyclohexane and potassium hydroxide were obtained from Fisher Scientific, Co. (Fair Lawn, NJ, USA). Absolute ethanol was obtained from Merck (Merck KGaA, Darmstadt, Germany). Campesterol, cholestane, cholesterol, stigmasterol, and sitosterol standards were obtained from Sigma-Aldrich, Inc. (Sigma Chemical, St. Louis, MO, USA). The trimethylsilyl (TMS) ether derivatives of all sterols were prepared using *N*,*O*-bis-[trimethylsilyl]trifluroacetamide reagent (Pierce Chemical Co., Rockford, IL, USA). 

### 2.2. Saponification and Derivatization

Tubers of *Diascorea alata* cv. Tainung No. 2 were cultivated and collected from Tao-Yuan, Taiwan. Yam tubers were cleaned and cut into 4-mm slices using a Salad Shooter (National Presto Industries, Eau Claire, WI, USA) without peeling. Heavy metal contents in the raw *Diascorea* materials were determined before the extraction processes (Supplementary Table S1). The preparation and extraction procedures of ground *Diascorea* powder have been described [13]. In brief, dried slices of *Diascorea* (50 g) were mixed with 500 mL de-ionized water (Milli-Q, Millipore Co., Bedford, MA, USA) at 45 °C for 1 h. The supernatant was collected and the sediment particles were again extracted for another three times using the same extraction procedure. The extracted supernatant was lyophilized at 50 °C to reach the final volume of 400 mL. These concentrated *Diascorea* extracts were ready for further analysis. Phytosterols were determined from *Diascorea* extracts by saponification with 1 M ethanolic KOH, heating at 80 °C for one hour, and extraction with hexane [16]. An internal standard (5α-cholestane) was added before saponification to correct for recovery and response of gas chromatography to phytosteroids. The unsaponifiable fraction was recovered by extraction with cyclohexane. The recovered fraction was transferred by pipette into tared glass vials and dried under nitrogen. TMS ether derivatives were prepared by dissolving 5-mg samples of the unsaponifiable materials into 1.0 mL of cyclohexane containing 50 μL of *N*,*O*-bis-[trimethylsilyl]trifluroacetamide reagent and heating the mixture for 1 h at 60 °C. The saponification procedure was performed in duplicate on aliquots of each lipid extract.

### 2.3. Phytosterol Analysis

Structural identifications and quantitative analyses of phytosterol derivatives were obtained using a Hewlett-Packard Agilent 6890/Agilent 5973 GC-MS ( Agilent Technologies, Santa Clara, CA, USA). Splitless injections were made with 1-μL sample volumes at an inlet temperature of 280 °C. Helium carrier gas flow was 1.0 mL/min with an average velocity of 43 cm/s. Separation was achieved with an HP-5MS capillary column (Agilent Technologies, Santa Clara, CA, USA) measuring 30-m × 250-μm ID × 0.25-μm film thickness. The oven temperature was programmed to begin at 170 °C, held for 1 min; increase to 300 °C at 15 °C/min; and held for 10 min. The total run time was 19.67 min. The mass selective detector was operated in scan mode over the range 40–500 m/z with the source heated to 230 °C and the quadrapole heated to 150 °C.

### 2.4. Quantification Analysis

The quantification analysis was conducted with a Hitachi-L 2130 HPLC pump (Hitachi Instruments Inc., Tokyo, Japan). Isocratic elution was performed by methyl alcohol and acetonitrile (10/90, *v*/*v*) as the mobile phase with flux rate of 1.2 mL/min. The Cosmosil RP-18 column (250-mm length × 4.6 mm i.d., 5-μm thickness) (Hitachi Instruments Inc., Tokyo, Japan) was used as the stationary phase. Hitachi L-2400 (Hitachi Instruments Inc., Tokyo, Japan) with a diode array detector was used to detect phytosterols. All spectra peaks were acquired with the temperature set at 21 ± 0.2 °C and a maximum wavelength absorption of 205 nm. Beta-sitosterol in a concentration of 200 μg/mL was used as the pure standard. Five grams of freeze-dried *Diascorea* powder was dissolved in 50 mL of 70% methanol for 3 h at 25 °C, followed by filtration and condensation in the rotary evaporator (Rotavapor RE111 Buchi Co., Flawil, Switzerland). The condensed standard β-sitosterol or *Diascorea* extract were injected at 20 μL into a Hitachi L-2200 spectrophotometer (Hitachi Instruments Inc., Tokyo, Japan) and the linear regression equation of the standard curves were obtained by plotting the amount injected against the peak area. The standard curve of β-sitosteroid was determined in triplicate and the mean value was calculated.

### 2.5. Design

This was a continuing analysis of a prospective, randomized, double-blind, placebo-controlled study carried out in two parallel groups of postmenopausal women. The details of the study design have been described previously [13]. To be enrolled, the women had to satisfy the following inclusion criteria: 45–60 years of age, physiological menopause within five years, no pharmacological treatment within 90 days prior to enrolment, serum follicle stimulating hormone (FSH) level >40 mIU/mL and cooperative ability. The exclusion criteria were the following: surgical and/or premature menopause, hypersensitivity to product ingredients, history of mammary, and/or genital malignant disease, presence of severe debilitating diseases, or cooperative inability. No patients recruited were on over-the-counter or other complimentary treatment during the investigation period. Recruited subjects were assigned, following a computer generated randomization list, to the *Diascorea* treatment or to placebo treatment. The study was carried out at Chang Gung Memorial Hospital Kaohsiung Medical Center and Tainan Hospital, Department of Health from February 2004 to December 2005. This study was carried out following the rules of the Declaration of Helsinki, the institutional review boards of both hospitals approved the study, and all participants provided written informed consent before study initiation. The pre-randomization screening and the preparation of *Diascorea*/placebo sachets have been described [13].

### 2.6. Intervention

The treatment posology was one sachet twice daily for 12 months of treatment. The product used in this study contains 12 mg of extracted *Diascorea* for each sachet. The dispensing of *Diascorea*/placebo and the compliance with the treatment in both groups of subjects was as described [13]. The primary efficacy parameters as plasma lipids profiles, antioxidant status, and hematological profiles, and secondary efficacy parameters including breast and uterus ultrasonographic scanning, were followed at six and 12 months’ treatment.

### 2.7. Experimental Methods 

Clinical safety parameters, including the measurements of plasma glucose and concentrations of liver and kidney enzymes (SGOT, SGPT, uric acid, and creatinine), were determined (Beckman Synchron CX7 Clinical Systems, Beckman Coulter, Inc., Brea, CA, USA).Various hematological parameters, including white blood cell (WBC) count, red blood cell (RBC) count, hemoglobin (Hb) concentration, hematocrit (Ht), mean corpuscular volume (MCV), mean cell hemoglobin (MCH), mean cell hemoglobin concentration (MCHC), and platelet count, were determined by using a Coulter Counter (Coulter MD series, Beckman Coulter Inc., Miami, FL, USA). Blood pressures were measured before and every month after *Diascorea* ingestion for 12 months. Bone mineral density on lumbar spine L1–L4 and bilateral femoral necks employing dual-energy X-ray absorptiometry were carried out at six and 12 months after ingestion of *Diascorea*. The details on the monitoring and adverse events were as described [13]. Malondialdehyde (MDA) in serum was assessed colorimetrically at 586 nm using a commercial kit (Cat.No. 437634, Calbiochem., Calbiochem-Novabiochem. Cor., La Jolla, CA, USA). The concentration of MDA expressed as nmol/mg protein was quantitatively estimated by the thiobarbituaric acid method, which resulted in profound lipid peroxidation inhibition by serum content. Total antioxidant status (TAS) was estimated using the Randox Total Antioxidant Status (TAS) Colorimetric kit (NX 2331 and 2332, Randox, Randox Laboratories Ltd., Antrim, UK). Total cholesterol (TC), triglycerides (TG), low-density lipoprotein cholesterol (LDL-C), and high-density lipoprotein cholesterol (HDL-C) of plasma were measured by using the timed-endpoint method. Apolipoprotein A-1 (Apo A-1) and apolipoprotein B (Apo B) were measured using turbidimetric method as described in manufacturer’s protocol (Beckman Coulter Inc. Brea, CA, USA). Ultrasonographic scanning of breast and uterus were carried out at six and 12 months after ingestion of *Diascorea*. The details on the monitoring and adverse events were as described [13].

### 2.8. Statistical Analysis

Changes from baseline for both primary and secondary efficacy parameters were compared between placebo and *Diascorea* treatment at two follow-up time points (month 6 and month 12). This study used one-way ANCOVA to analyze these data. For each variable, the covariate was the value at month 0 and the fixed factor was the treatment type (*Diascorea* and placebo). Dependent variables are the value at six months and 12 months, respectively, for plasma antioxidant activities, lipid profiles, hematological profiles, and other efficacy parameters. If the model was significant, then the treatment would be checked for significance or not. In all cases, the differences were considered significant at *p* < 0.05. Data were expressed as mean values (standard deviation) when normally distributed. All data were analyzed using SPSS version 18.0 software (IBM, Chicago, IL, USA). As mentioned previously [13], a sample size of 25 individuals in each group was acceptable for the analysis of covariance to maintain the power of a study with a two-tailed value of 0.05.

## 3. Results

Analysis of the corresponding TMS derivatives by GC-MS revealed a pattern of one major peak, tentatively identified as β-sitosterol in the total ion chromatogram (Supplementary Figure S1). Structural identification was confirmed by comparison of the retention times and mass spectra obtained for the *Diascorea* sample and those of derivative sterol standards. Beta-sitosterol, 22-23-dihydro-stigmasterol, and γ-sitosterol were major compounds determined with recovery rates of 94%, 88%, and 86%, respectively (Supplementary Figure S2). The retention time of these compounds in this determination was 14.61 min. Few minor peaks identified tentatively represent hexadecane, octadecane, 9-ethyl-9-heptyl-octadecane, and 2-ethylacridine. The unsaponified fractions obtained from 5.0004 g of *Diascorea* root extracts were determined to contain 530 μg of β-sitosterol. This was comparable to the sterol content of 106 μg of sterol/gram of crude weight of wet unextracted *Diascorea* root.

### Participants and Follow-Up

Initially, 164 women were approached and 114 women were excluded from this study (Supplementary Figure S3). The demographic parameters at baseline for the two groups of 50 postmenopausal women were comparable [13]. The baseline values of blood pressure, liver and renal functions, plasma glucose, and lipid profiles were within normal limits in both groups. The hematological profiles were also within normal limits and were all comparable at baseline in both groups of subjects, except higher WBC counts in *Diascorea* group.

**Figure 1:**
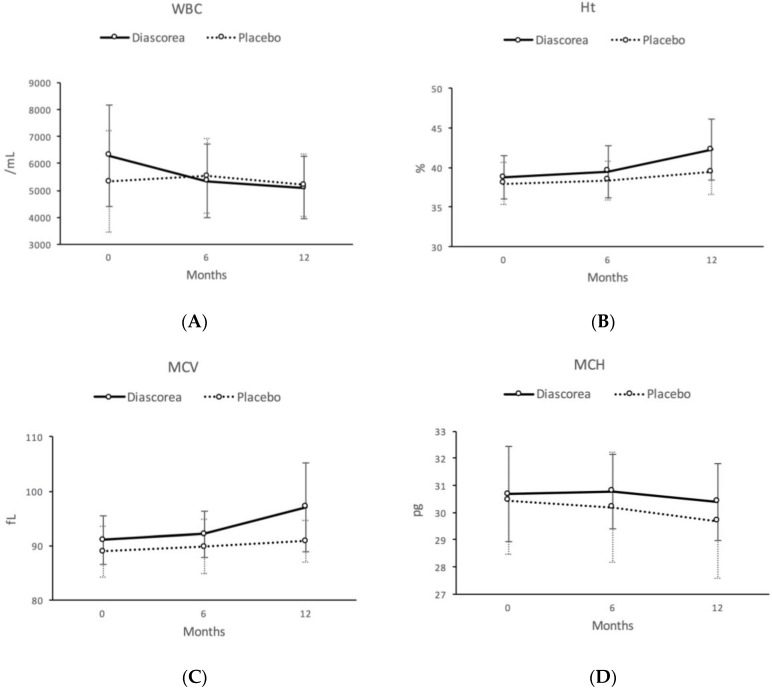
The change patterns of WBC (**A**), Ht (**B**), MCV (**C**), and MCH (**D**) between Month 0, Month 6, and Month 12.

At six months in those receiving *Diascorea*, there were significantly decreased WBC counts (Table 1). A transient improvement in antioxidant activity of MDA (*p* < 0.001) was found and this phenomenon did not persist to the 12 month treatment point (Table 2). After 12 months of treatment, elevations of Ht and MCV were noted in those taking *Diascorea* (Table 1, Figure 1). Reduced levels of MCH were noted in the placebo group, but not in those taking *Diascorea*. Though not approaching a significant difference, there is a trend of increased Hb level and RBC counts in the *Diascorea* group during this trial. There was a similar trend in the reduction of cholesterol and HDL-C in the *Diascorea* and placebo group, respectively. The LDL-C was increased in both groups. The TG concentrations were reduced in *Diascorea* group, but did not reach a significant difference compared to the placebo group. The serum concentrations of ApolipoA1 and ApolipoB were slightly reduced in those receiving *Diascorea* (Table 2). However, all those serum lipid profiles generally did not reach significant differences in the treatment. No changes of bone mineral density were noted in the present study.

After 12 months of treatment, the BMI in the *Diascorea* group was significantly decreased (Table 1). There was no specific change on the hepatic and renal functions for those women receiving *Diascorea* for one year. Only three women presented transient gastroenterologic symptoms, such as bloating and constipation, during the study period, and no serious adverse events or harmful complications led to discontinuation in the investigation. Though there was a tendency of reduced number and size of breast cysts in women of *Diascorea* group, it did not reach a significant difference (Table 2).

## 4. Discussion

### 4.1. Main Findings

In the present study, phytosterols have been identified as the major components in *Diascorea alata*. One-year intake of *Diascorea alata* extracts showed no adverse effects relevant to the liver and renal functions in postmenopausal women. Although there was no significant effect on plasma lipid profiles, modulating effects on blood components and transient improvement of antioxidant activity by low dosage of *Diascorea* were found. This study also showed that low-dose ingestion of *Diascorea* extracts might reduce the number and size of breast cysts.

### 4.2. Aging and Hematopoiesis

Menopause, the cessation of ovulation, indicates the start of an abrupt aging process in women. The hematopoiesis may become exhausted in postmenopausal women due to progressive loss of stem cells and of their renewal capacity as aging [6,17,18]. Studies indicated that older humans are less able than young ones to mobilize neutrophiles after injection of hydrocortisone and to increase the concentration of hematopoietic progenitors in the circulation after injections of growth factors [19,20]. Only small-scale investigation of hematological profiles in postmenopausal women has ever been reported [21,22]. Some blood cell components, especially Hb, were higher in postmenopausal women [23,24], and the reasons for the difference were related to the effects of the hormonal environment of menopause [25].

### 4.3. Effects of Phytosterols/Diascorea on Blood Cells in Menopause

In the present study, the baseline RBC counts and Hb levels were at lower levels of the adult values, indicating the presence of mild anemia in Taiwanese postmenopausal women. Though not reaching a significant difference, the indices of RBC count and Hb were elevated in those receiving *Diascorea* ingestions for 12 months. The study indicated that the increase in red cell indices is usually noticed in women who were at least 10 years postmenopausal [26]. The mean age of our subjects was 51.92 and 53.08 years old, and the interval from menopause to the study was 2.64 and 2.56 years in *Diascorea* and control groups, respectively [13]. Thus, the ingestion of *Diascorea* might enhance the hematopoiesis in postmenopausal women. Recently, β-sitosterol has been shown to decrease the nuclear translocation of nuclear factor kappa B (NF-κB) [27,28]. NF-κB consists of five members—p65(RelA), RelB, c-Rel, p50/p105(NF-κB1), and p52/p100(NF-κB2)—and their signaling in hematopoietic differentiation has been extensively reviewed [27]. In a mouse study, lacking RelA/p65 in the hematopoietic compartment severely impairs HSC function and occurs in conjunction with increased hematopoietic stem and progenitor cell cycling, extramedullary hematopoiesis, and differentiation defects [29]. An animal study also indicated that RelB/NF-κB2 signaling positively and intrinsically regulates hematopoietic stem/progenitor cell self-renewal and maintains stromal/osteoblastic niches, and negatively and extrinsically regulates hematopoietic stem/progenitor cell expansion and lineage commitment through the marrow microenvironment [30]. Thus, the NF-κB inhibited by β-sitosterol might play an important role in the bone marrow hematopoietic microenvironment, which then modulates the hematopoiesis.

In the present study, the MCV and Ht in postmenopausal women before the test was in the normal range. After receiving *Diascorea* treatment, MCV and Ht levels were significantly elevated (*p* < 0.01). The increase of the MCV in the postmenopausal women has been observed [31]. The study indicated that almost every parameter of HSC function was altered in some way during the aging process and may lead to functional exhaustion in a permissive genetic background [32]. The qualitative changes of hematopoiesis may be related to alteration of the genome from environmental substances and nutritional deficiency, psychological stress, endocrine changes, oxidative stress, and disruption of the hematopoietic microenvironment [32]. Significant improvement of the psychological scales and hormone status in postmenopausal women ingestion of *Diascorea* has been found in our previous report [13]. Enhanced antioxidant activity has also been noted in the present study. In addition to the modulation of NF-κB signaling in the hematopoietic differentiation by β-sitosterol mentioned above, the effect of *Diascorea* ingestion on qualitative changes of hematopoiesis needs further investigation. In an animal study, a decrease in Ht was observed during egg production in birds, which might be due to antagonistic pleiotropic effects of estrogen [33]. We have shown elevated serum estradiol and DHEAS levels, and decreased FSH concentration in those who ingested *Diascorea* [13]. Whether the alteration of Ht was relevant to the ingestion of *Diascorea* and its content of phytosterol needs further study.

Although significant changes in the WBC counts were noted in the study group, the relatively higher baseline WBC counts in those women might cause a bias in the present study. One drawback of the present study was that we did not analyze the differential counts of leukocytes, thus, we might not be able to identify whether the alterations were mostly on lymphocytes or neutrophiles. Lymphocytopenia has been shown in postmenopausal women who received a synthetic isoflavone for three years in a multicenter study [34]. However, a recent study found that isoflavone for one year did not alter lymphocyte counts in postmenopausal women [35]. Thus, the impact of HSCs and lymphopoiesis by plant components of isoflavoneor phytosterols remains to be solved.

### 4.4. Lipid Profiles in Postmenopausal Women

Epidemiological studies suggest that menopause is associated with increases in CVD risk factors with incidence markedly increased after the ages of 45 to 54 years [36]. Higher levels of TC, LDL-C, TG, and Apo B were found in post-menopausal, compared to pre-menopausal, women, while inconsistent results were found for HDL-C [37,38]. Recent results from the Study of Women’s Health Across the Nation (SWAN) showed considerable increases in levels of TC, LDL-C, and TG in the one-year interval before and after the final menstrual period that are consistent with menopause-induced changes [39]. In addition, the greatest increase in HDL-C and Apo A1 levels was observed in the late perimenopausal period [39]. Increased prevalence of lipid and lipoprotein abnormalities were observed in a cross-sectional analysis of 1553 Korean women aged 44–56 years, across menopausal stages [40].

### 4.5. The Effects of Phytosterols on Lipid Profiles

Phytosterols has been shown to reduce the plasma cholesterol and presented as cardioprotective components [41,42]. Numerous trials have confirmed the efficacy of phytosterolsin reducing LDL-C, without significantly altering HDL-C or TG [43,44]. A meta-analysis of 14 clinical trials showed that plant sterol consumption of 0.8–4.0 g/day led to reductions in LDL-C concentrations of 0.33–0.54 mmol/L [45]. Another meta-analysis of 26 clinical trials, a measurable LDL-C lowering effect was observed with phytosterol intakes of 900 mg/day [46]. Dosages ranging from 0.8 to 3.0 g/day of plant sterols and stanols have been used to produce cholesterol reductions [47]. Very small intakes of 150 and 300 mg of phytosterols added to sterol-free corn oil in a single test meal reduced cholesterol absorption by 12.1% and 27.9%, respectively [46]. More recent work showed micellarphytosterols reduced cholesterol absorption by 23.3% and 32.0% at a dose of 300 mg and 500 mg, respectively [48]. These cholesterol-lowering properties of phytosterols have been attributed to the inhibition of cholesterol absorption resulting from the higher affinity of phytosterols than of cholesterol for micelles [49] and enhanced stimulation of the ATP-binding cassette transporter G or greater displacement of cholesterol from micelles within the intestinal lumen [50]. However, controversial results from a recent systematic review and meta-analysis did not reveal any evidence of an association between serum concentrations of plant sterols and the risk of CVD [51]. Data from the present study did not show significant changes on lipid profiles in our subjects. We suggest that the low dose of *Diascorea* with phytosterol content used in this study may explain the finding as the content of phytosterol in this study was only about 1–2 mg/day, a dose much lower than what was used in all previous studies. 

### 4.6. Modulating Antioxidant Activity by Diascorea/Phytosterols

Phytosterolhas been shown to suppress the oxidation and consumption of α-tocopherol in β-linoleoyl-γ-palmitoylphosphatidylcholine liposomal membranes [52]. Study showed that β-sitosterol modulated both glutathione peroxidase as well as superoxide dismutase levels and reverted the impairment of the glutathione/oxidized glutathione ratio induced by phorbol esters in RAW 264.7 macrophage cultures [53]. Changes in plasma lipoprotein levels with a decrease of oxidized LDL has been demonstrated in subjects whose diet was supplemented with 2 g/day of plant sterols, suggested an antioxidant effect of plant sterols [54]. Recent study showed the depletion of cellular glutathione was effectively inhibited by the galloylphytosterol antioxidant [55]. Estrogens were found to act as antioxidants via the stimulation of antioxidant enzymes [56]. Binding affinity of β-sitosterol for membrane estrogen receptors has been shown in macrophage [57]. A regulatory pathway, mediated by membrane estrogen receptor and PI3-kinase activation has been identified, through which β-sitosterol may reduce cellular ROS levels [53]. Besides, reduced NF-κB activation by phytosterols might also be used to explain the antioxidant effects [28,58]. The present study shows that a low dose of phytosterol acts as an antioxidant, transiently. Further study is needed to investigate why this effect could not last throughout the 12 months’ study. Recent study indicated the phytosterols’ content and antioxidant activity from plants could be changed with different solvents used for extraction, e.g., the phytosterols and antioxidant values were significantly higher in ethanol extract, while extract from hexane had a significantly higher yield of extract, and lower oxidative content [59]. The solvent used for extraction of phytosterols in the present study was hexane and might result in lower oxidative content.

A recent study suggested that *Diascorea alata* ingestion decreased the metabolism of estrogens and, especially, the formation of the carcinogenic metabolite 16α-hydroxyestrone [12]. In addition to the possible correlation between estrogen metabolites and downregulation of NF-κB, the role of improvement in the antioxidant status after the administration of *Diascorea* relevant to the reduced number and breast cyst size in the present study needs further investigation.

### 4.7. Strengths and Limitations

This randomized controlled study on human subjects employing the plant extract from *Diascorea alata* containing phytosterols provides evidence that low-dose intake of *Diascorea*/phytosterols may improve the antioxidant status and hematological profiles in postmenopausal women. The identification of active ingredients as phytosterols in *Diascorea* could provide information for menopausal women to choose this plant component for alternative treatment. Beneficial effects on menopausal symptoms, modulation of hematological profiles, antioxidant activities, and other physiological functions, as well as no adverse reactions, further provide reassurance on the safe usage of this plant product. The major drawback of the study was the small number of subjects recruited and the relatively short time period. Another limitation of the present study was that the serum non-cholesterol sterol levels were not determined in correlation to the dietary phytosterol intake. Further pharmacokinetics and pharmacodynamics studies should help to demonstrate that different ingredients of *Diascorea*, including phytosterols absorbed through the digestion system, exert clinical functions in the human body.

Additionally, the active ingredients in *Diascorea alata* may be far more than phytosterols, e.g., anthocyanin, allantoin, and dioscorin in *Dioscorea purpurea* have been identified [60]. Dioscorin, yam storage protein, has been demonstrated with scavenging properties against free radicals, immunomodulatory activity, as well as antihypertensive activities in rats [61,62]. Diosgenin and dioscin, the steroidal saponins in *Diascorea*, showed estrogenic, antioxidant, anti-inflammatory, and anti-tumor effects [63]. Thus, future studies may use individual *Diascorea* components in further investigations. Recent studies indicated an increase in fibrosis in the kidneys and inflammation in livers of rats consuming *Diascorea villosa* for 28 days [64,65]. Thus, further usage of *Diascorea* in humans must be cautious on this issue. Though not significantly different, the tendency of decreased breast cyst number and size after intake of *Diascorea* may shed light on further prevention of the occurrence of breast cancer.

## 5. Conclusions

The present study identified that β-sitosterol is the major phytosterol in *Diascorea alata Alata*. Our clinical data showed that *Diascorea alata Alata* intake had a beneficial impact on the hematological and antioxidant activities in postmenopausal women. The positive outcome and safety record observed in this study suggests that postmenopausal women can use *Diascorea alata* as a safe and effective botanical supplement. A larger study will be needed to confirm our findings.

## Figures and Tables

**Table 1:** The effects of *Diascorea* on hematological profiles after six and 12 months. Data are given as the mean (standard deviation).

	*Diascorea* (*n* = 25)						Placebo (*n* = 25)						*F* (0 and 6)		*F* (0 and 12)	
	Month 0		Month 6		Month 12		Month 0		Month 6		Month 12					
Body weight (kg)	56.40	(8.32)	56.40	(8.22)	55.80	(8.03)	58.85	(8.24)	58.60	(8.46)	58.65	(8.54)	0.98		0.37	
BMI (kg/m^2^)	23.37	(3.93)	23.12	(3.59)	22.81	(3.16)	24.19	(3.13)	23.95	(2.99)	24.20	(3.24)	0.22		6.69	*
Systolic blood pressure (mmHg)	118.68	(13.48)	111.92	(12.11)	113.12	(14.72)	125.16	(18.67)	119.76	(13.43)	116.12	(13.96)	2.59		0.12	
Diastolic blood pressure (mmHg)	74.68	(9.75)	73.72	(10.92)	73.56	(11.08)	74.52	(15.22)	72.36	(10.45)	71.48	(11.57)	0.36		0.51	
Glucose fasting (mg/dL)	82.48	(6.47)	76.80	(4.76)	90.36	(8.06)	99.40	(18.77)	84.28	(19.73)	94.56	(28.36)	0.19		2.78	
Uric acid (mg/dL)	5.04	(1.35)	5.41	(1.39)	5.08	(1.46)	5.43	(1.49)	5.70	(1.30)	5.41	(1.48)	0.01		0.02	
Creatinine (mg/dL)	0.78	(0.08)	0.78	(0.07)	0.74	(0.09)	0.84	(0.13)	0.84	(0.10)	0.80	(0.10)	2.19		1.30	
SGOT (U/L)	25.68	(6.45)	26.64	(6.30)	21.96	(5.96)	27.24	(7.89)	25.56	(5.93)	25.28	(10.05)	1.58		1.39	
SGPT (U/L)	18.68	(5.61)	12.56	(6.41)	18.48	(5.39)	20.88	(9.62)	16.68	(6.92)	23.44	(13.77)	3.65		1.97	
BMD (g/cm^2^)	0.91	(0.15)	0.90	(0.15)	0.89	(0.15)	0.95	(0.14)	0.94	(0.14)	0.94	(0.14)	1.08		3.89	
WBC (/mL)	6288.0	(1870.5)	5352.0	(1380.9)	5098.8	(1151.6)	5322.4	(1200.1)	5536.0	(1235.8)	5200.0	(1208.9)	14.22	**	5.49	*
RBC (10^9^/dL)	426.68	(37.46)	429.88	(43.46)	436.92	(42.82)	428.48	(41.07)	428.68	(39.36)	435.60	(43.59)	0.33		0.28	
Hb (g/dL)	13.05	(0.92)	13.20	(1.08)	13.23	(0.90)	13.00	(1.05)	12.90	(0.96)	12.87	(0.91)	2.22		3.59	
Ht (%)	38.73	(2.73)	39.48	(3.27)	42.25	(3.89)	38.00	(2.72)	38.36	(2.46)	39.44	(2.88)	1.00		8.57	**
MCV (fL)	91.01	(4.49)	92.09	(4.23)	97.08	(8.23)	88.96	(4.64)	89.79	(5.03)	90.80	(3.90)	0.52		10.13	**
MCH (pg)	30.68	(1.76)	30.78	(1.38)	30.40	(1.41)	30.44	(1.98)	30.20	(2.03)	29.69	(2.10)	3.84		6.30	*
MCHC (g/L)	33.70	(0.82)	33.44	(0.79)	31.45	(2.07)	34.20	(1.03)	33.64	(1.00)	32.67	(1.36)	1.15		2.70	
Platelet (10^9^/L)	24.54	(4.78)	23.07	(4.35)	23.50	(5.04)	23.42	(4.62)	24.25	(4.52)	22.98	(4.69)	6.73	*	0.23	

* *p* < 0.05; ** *p* < 0.01. SGOT, serum glutamic oxaloacetic transaminase; SGPT, serum glutamic pyruvic transaminase.

**Table 2:** The effects of *Diascorea* on antioxidant status, lipid profiles, and breast cysts after six and 12 months. Data are given as the mean (standard deviation).

	*Diascorea* (*n* = 25)						Placebo (*n* = 25)						*F* (0 and 6)		*F* (0 and 12)
	Month 0		Month 6		Month 12		Month 0		Month 6		Month 12				
TAS (mmole/L)	8.56	(4.87)	10.64	(6.67)	10.39	(6.36)	11.65	(5.49)	9.22	(5.17)	9.09	(4.76)	2.36		2.51
MDA (nmol/mg)	36.03	(8.19)	18.81	(4.25)	12.49	(2.31)	30.95	(10.44)	14.58	(3.46)	12.92	(2.03)	16.47	***	0.16
Cholesterol (mg/dL)	212.28	(36.58)	213.28	(38.05)	200.88	(36.84)	229.16	(38.41)	222.08	(31.62)	215.48	(37.15)	0.15		0.06
Triglyceride (mg/dL)	159.00	(138.82)	144.12	(134.39)	118.0	(82.52)	123.56	(78.51)	131.64	(73.64)	123.80	(66.43)	0.73		2.23
HDL (mg/dL)	68.60	(15.82)	56.73	(12.44)	55.31	(10.80)	72.12	(20.55)	58.81	(15.78)	59.46	(18.06)	0.07		0.51
VLDL (mg/dL)	19.02	(13.93)	14.83	(12.54)	9.12	(5.80)	17.77	(18.53)	16.26	(10.54)	9.36	(5.29)	0.54		0.13
LDL (mg/dL)	124.65	(21.78)	141.74	(27.23)	136.44	(33.78)	139.39	(26.74)	147.00	(23.83)	146.68	(37.07)	0.77		0.49
T.CHO/HDL	3.21	(0.72)	3.90	(0.98)	3.74	(0.95)	3.40	(1.14)	4.01	(1.18)	3.94	(1.39)	0.00		0.03
LDL/HDL	1.90	(0.50)	2.60	(0.69)	2.56	(0.84)	2.08	(0.71)	2.69	(0.90)	2.75	(1.25)	0.01		0.00
Apolipo.Al (mg/dL)	162.26	(28.84)	153.68	(14.63)	152.52	(15.08)	159.06	(44.69)	159.64	(25.63)	152.18	(24.21)	1.53		0.01
Apolipo.B (mg/dL)	95.04	(21.84)	100.45	(21.00)	87.60	(20.24)	99.70	(23.75)	105.37	(24.12)	94.28	(26.18)	0.12		0.51
Number of breast cyst	1.04	(1.17)	0.52	(1.05)	0.76	(1.30)	0.20	(0.41)	0.12	(0.33)	0.32	(0.85)	0.72		0.45
Total size of breast cyst (cm)	0.61	(0.75)	0.34	(0.80)	0.39	(0.74)	0.11	(0.24)	0.06	(0.15)	0.17	(0.49)	0.42		0.81

* *p* < 0.05; ** *p* < 0.01; *** *p* < 0.001. SGOT, serum glutamic oxaloacetic transaminase; SGPT, serum glutamic pyruvic transaminase.

## Supplementary Materials

The followings are available online at www.mdpi.com/2072-6643/9/12/1320/s1, Figure S1: GC-MS chromatogram for extract of *Diascorea alata* and structural identifications and quantitative analyses of phytosterol derivatives are presented, Figure S2: Structural identification was confirmed by comparison of the retention times and mass spectra obtained for *Diascorea alata* (upper figure) and those of derivative sterol standards of β-sitosterol, γ-sitosterol, and 22-23-dihydro-stigmasterol, Figure S3: Flow chart for recruiting postmenopausal women in this study, Table S1: The detection of retained heavy metals in *Diascorea* used.
