# Supplementary material for: The Effects of Phytosterols Extracted from Diascorea alata on the Antioxidant Activity, Plasma Lipids, and Hematological Profiles in Taiwanese Menopausal Women
Source: Nutrients. 2017 Dec 5;9(12):1320. doi: 10.3390/nu9121320 (PMC5748770; doi:10.3390/nu9121320)
Supplement: Supplementary file 1 [file nutrients-09-01320-s001.pdf]

Supplementary Figure Legends:

Supplementary Figure S1. GC-MS chromatogram for extract of *Diascorea alata* and structural identifications and quantitative analyses of phytosterol derivatives are presented. The peaks were identified by their retention times using standards. The areas under the peaks were measured with an integrator. Data were acquired and processed with Chemstation software. The upper and middle figures indicate the retention time of standard β-sitosterol and sample compounds in this determination was 14.61 minutes. The lower figure shows the chromatogram and structure of β-sitosterol.

Supplementary Figure S2. Structural identification was confirmed by comparison of the retention times and mass spectra obtained for *Diascorea alata* (upper figure) and those of derivative sterol standards of β-sitosterol, γ-sitosterol, and 22-23-dihydro- stigmasterol.

Supplementary Figure S3. Flow chart for recruiting postmenopausal women in this study.

Supplementary figure S1

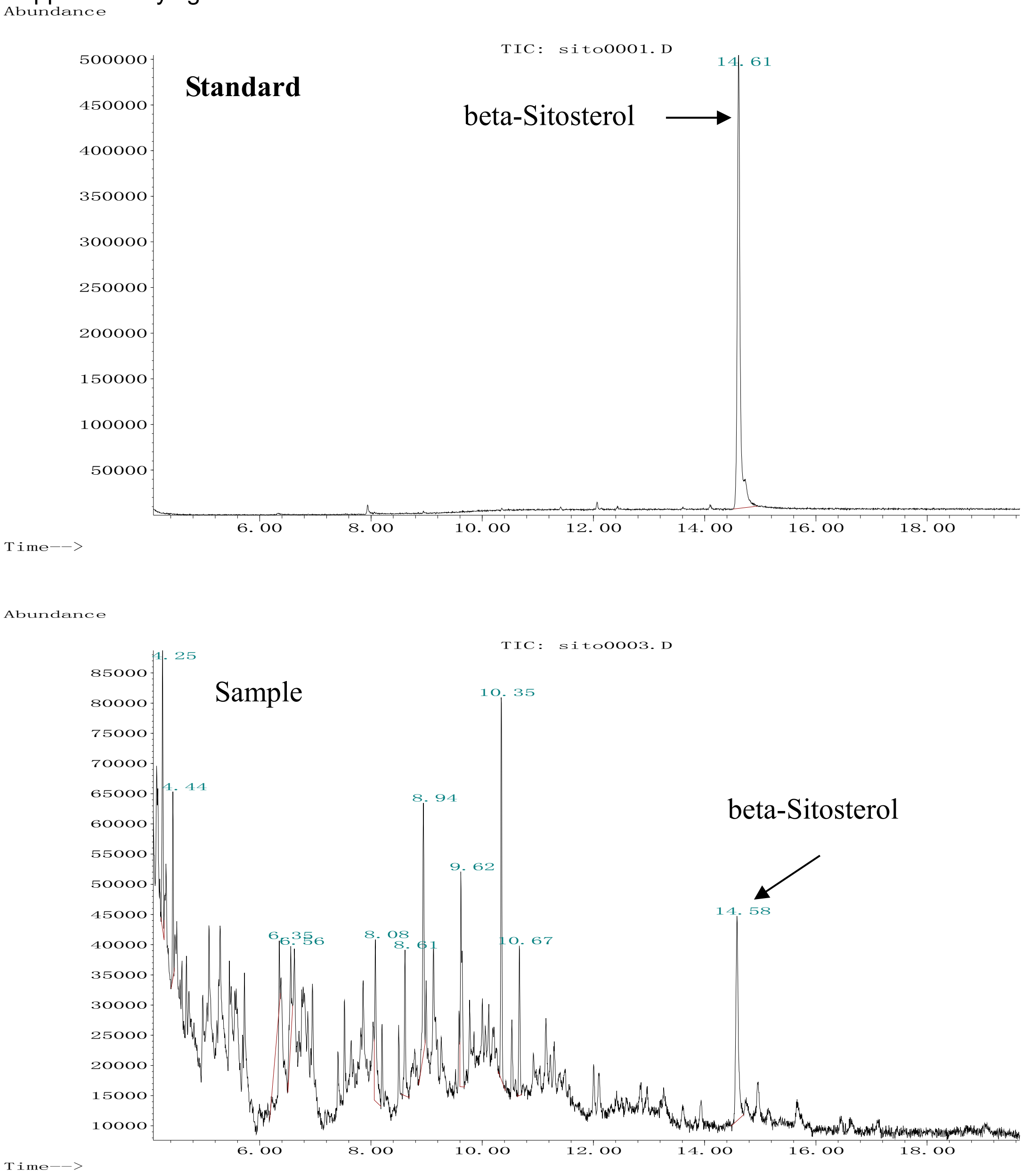

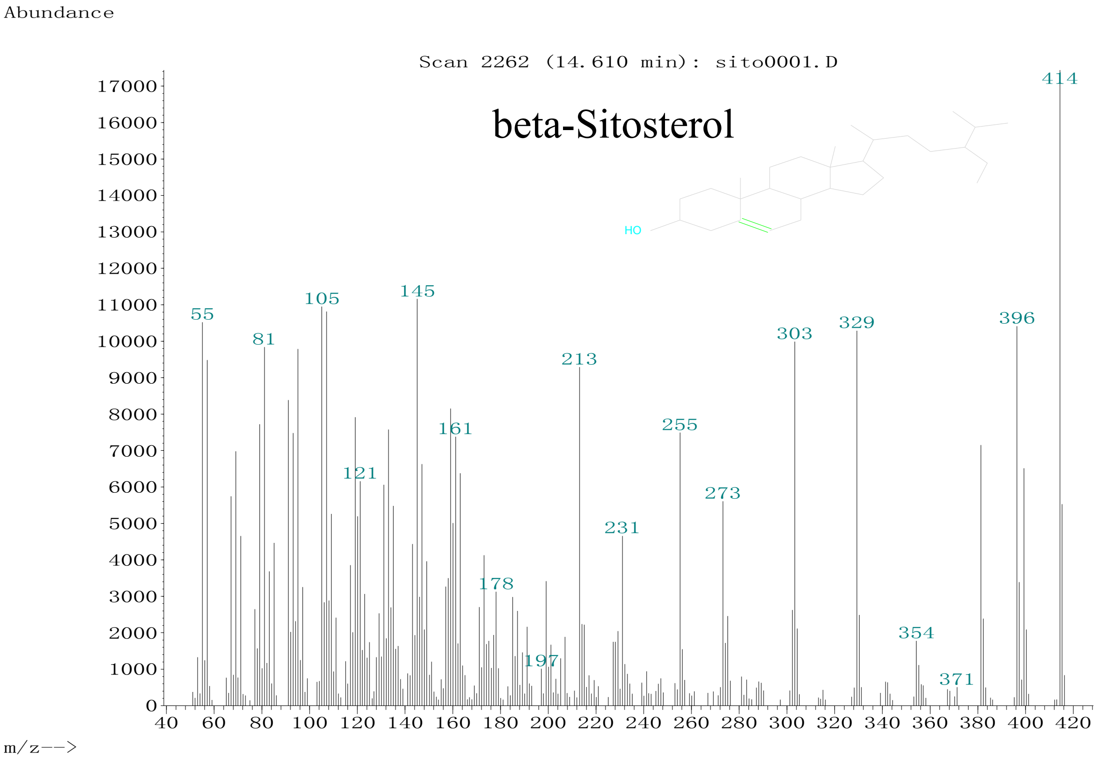

Abundance
Scan 2262 (14.610 min): sito0001.D
beta-Sitosterol
HO
17000
16000
15000
14000
13000
12000
11000
10000
9000
8000
7000
6000
5000
4000
3000
2000
1000
0
414
55
105
145
81
396
329
303
213
161
255
121
273
231
178
197
354
371
40 60 80 100 120 140 160 180 200 220 240 260 280 300 320 340 360 380 400 420
m/z-->

Supplementary figure S2

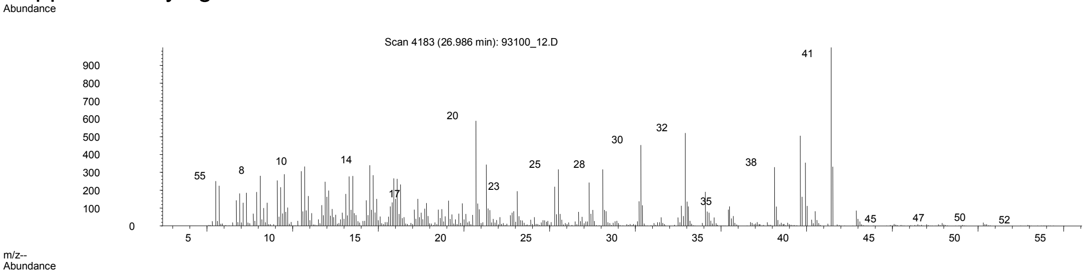

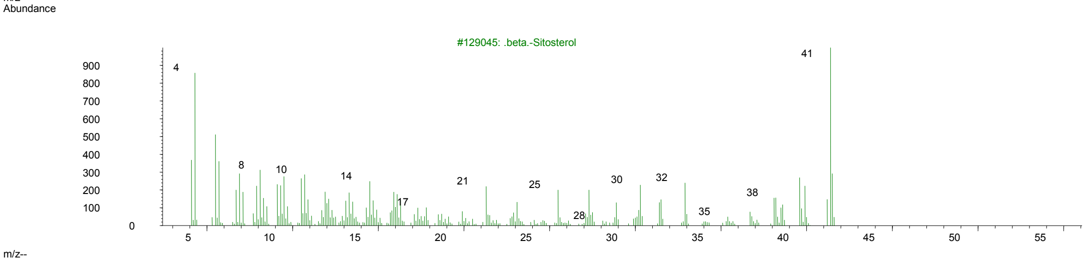

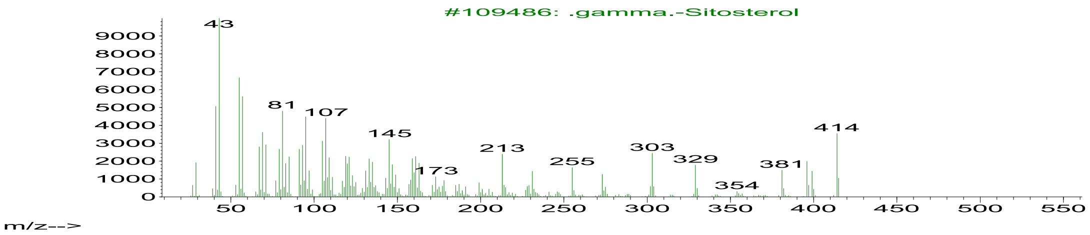

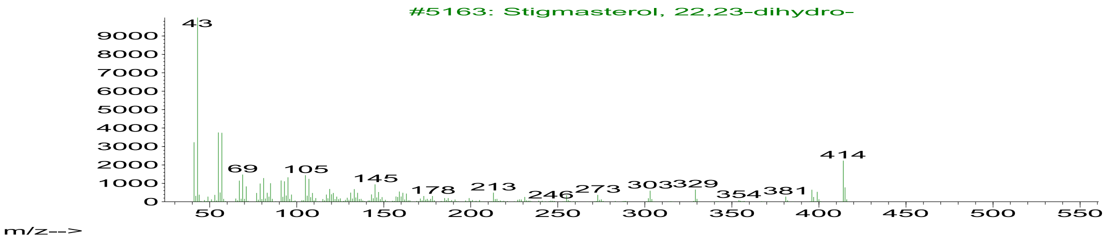

Supplementary figure S3

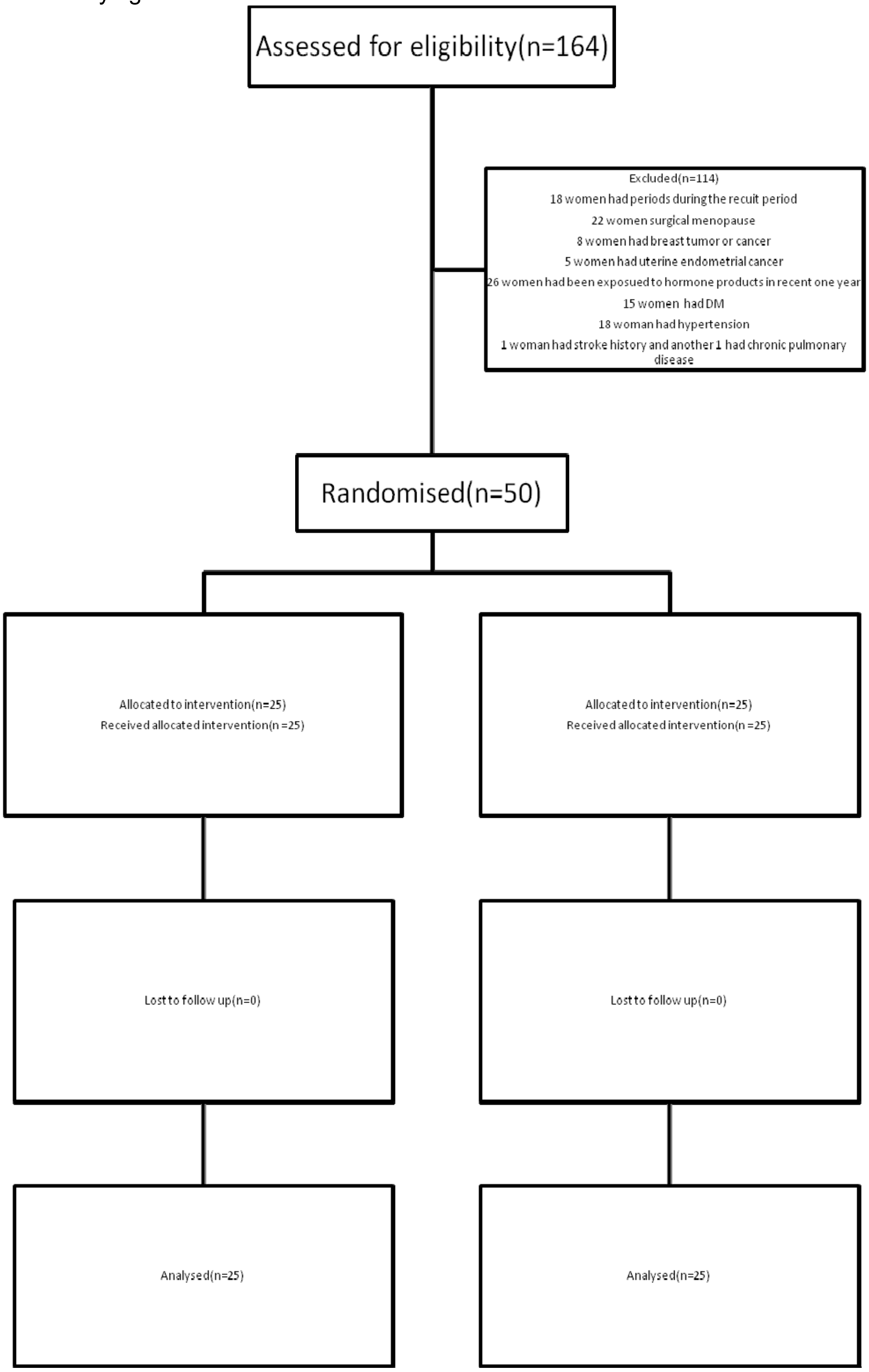

Supplementary Table S1. The detection of retained heavy metals in *Diascorea* used.

Heavy metals	(ppm)*	Unpeeled (ppm)	Peeled (ppm)
Pb***	<20ppm	ND**	ND
Cu	<100ppm	8.943ppm	6.04ppm
Cd	<1ppm	0.248ppm	ND
Hg	<0.5ppm	ND	ND
As	<5ppm	ND	ND

*: Tolerance level of heavy metal contents.

**: Non-detectable.

***: Pb, Cu, Cd, Hg, and As: lead, copper, cadmium, mercury, and arsenic.
